# Optimal choice of word length when comparing two Markov sequences using a *χ*^2^-statistic

**DOI:** 10.1186/s12864-017-4020-z

**Published:** 2017-10-03

**Authors:** Xin Bai, Kujin Tang, Jie Ren, Michael Waterman, Fengzhu Sun

**Affiliations:** 1 Centre for Computational Systems Biology, School of Mathematical Sciences, Fudan University, Shanghai, China; 2 Molecular and Computational Biology Program, University of Southern California, Los Angeles, California USA

**Keywords:** Markov chain, Alignment-free genome comparison, Statistical power, NGS

## Abstract

**Background:**

Alignment-free sequence comparison using counts of word patterns (grams, *k*-tuples) has become an active research topic due to the large amount of sequence data from the new sequencing technologies. Genome sequences are frequently modelled by Markov chains and the likelihood ratio test or the corresponding approximate *χ* ^2^-statistic has been suggested to compare two sequences. However, it is not known how to best choose the word length *k* in such studies.

**Results:**

We develop an optimal strategy to choose *k* by maximizing the statistical power of detecting differences between two sequences. Let the orders of the Markov chains for the two sequences be *r*_1_ and *r*_2_, respectively. We show through both simulations and theoretical studies that the optimal *k*= max(*r*_1_,*r*_2_)+1 for both long sequences and next generation sequencing (NGS) read data. The orders of the Markov chains may be unknown and several methods have been developed to estimate the orders of Markov chains based on both long sequences and NGS reads. We study the power loss of the statistics when the estimated orders are used. It is shown that the power loss is minimal for some of the estimators of the orders of Markov chains.

**Conclusion:**

Our studies provide guidelines on choosing the optimal word length for the comparison of Markov sequences.

**Electronic supplementary material:**

The online version of this article (doi:10.1186/s12864-017-4020-z) contains supplementary material, which is available to authorized users.

## Background

The comparison of genome sequences is important for understanding their relationships. The most widely used methods are alignment based algorithms such as the Smith-Waterman algorithm [1], BLAST [2], BLAT [3], etc. In such studies, homologous genes among the genomes are identified, aligned, and then their relationships inferred using phylogenetic analysis tools to obtain gene trees. A consensus tree combining the gene trees from all the homologous genes is used to represent the relationship among the genomes. However, non-conserved regions form large fractions of most genomes and they also contain information about the relationships among the sequences. Most alignment based methods do not consider the non-conserved regions resulting in loss of information. Another drawback of the alignment based method is the extremely long time needed for the analysis, especially when the number of genome sequences is large.

With the development of new sequencing technologies, a large number of genome sequences are now available and many more will be generated. To overcome the challenges facing alignment based methods for the study of genome sequence relationships, several alignment-free sequence comparison methods have been developed as reviewed in [4, 5]. Most of the methods use the counts of word patterns within the sequences [6–12]. One important problem is the determination of word length used for the comparison of sequences. Several investigators addressed this issue using simulation studies or empirical data [13–15]. Wu et al. [15] investigated the performance of Euclidian distance, standardized Euclidian distance, and symmetric Kullback–Leibler discrepancy (SK-LD) for alignment free genome comparison. For a given dissimilarity measure, Wu et al. [15] simulated the evolution of two sequences with different mutation rates and chose the word length that yielded the highest Spearman correlation between the dissimilarity measure and the mutation rate. They showed that SK-LD performed well and the optimal word length increases with the sequence length. Using a similar approach, Forêt et al. [14] studied the optimal word length for *D*_2_ that measures the number of shared words between two sequences [8]. Sims et al. [13] suggested a range for the optimal word length using alignment-free genome comparison with SK-LD.

Markov chains (MC) have been widely used to model molecular sequences to solve several problems including the enrichment and depletion of certain word patterns [16], prediction of occurrences of long word patterns from short patterns [17, 18], and the detecting of signals in introns [19]. Narlikar et al. [20] showed the importance of using appropriate Markov models on phylogenetic analysis, assignment of sequence fragments to different genomes in metagnomic studies, motif discovery, and functional classification of promoters.

In this paper, we consider the comparison of two sequences modelled using Markov chains [11, 12] as a hypothesis testing problem. The null hypothesis is that the two sequences are generated by the same Markov chain. The alternative hypothesis is that they are generated by different Markov chains. We investigate a log-likelihood ratio statistic for testing the hypotheses and its corresponding *χ* ^2^-statistic based on the counts of word patterns in the sequences. The details of the statistics are given in “The likelihood ratio statistic and the *χ*^2^-statistic for comparing two Markov sequences” subsection. We use statistical power of the test statistic under the alternative hypothesis to evaluate its performance. We will study the following questions. a) What is the optimal word length *k* yielding the highest power of the *χ* ^2^-statistic? b) How do the estimated orders of the Markov sequences, sequence length, word length, and sequencing error rate impact the power of the *χ* ^2^-statistic? c) For NGS read data, what is the distribution of the *χ* ^2^-statistic under the null hypothesis? (d) Do the conclusions from (a) and (b) still hold for NGS reads?

## Methods

### Alignment-free comparison of two long Markov sequences

We study alignment-free comparison of two long Markov sequences using counts of word patterns. We first introduce the likelihood ratio [11, 12] and corresponding *χ* ^2^-statistic. We show theoretically and by simulations that the optimal word length is *k*= max{*r*_1_,*r*_2_}+1, where *r*_1_ and *r*_2_ are the orders of the two Markov sequences. We then study the effects of sequence length, word length, and estimated orders of MCs on the power of the *χ* ^2^-statistic.

### The likelihood ratio statistic and the *χ*^2^-statistic for comparing two Markov sequences

Given two Markov sequences **A**_1_ and **A**_2_, we want to test if the two sequences follow the same MC, that is, if their transition probability matrices are the same. We formulate this as a hypothesis testing problem. The null hypothesis *H*_0_ is that the two sequences are generated from the same MC. The alternative hypothesis *H*_1_ is that the two sequences are generated from MCs with different transition probability matrices.

To test the hypotheses, we use a likelihood ratio test statistic. Since we may not know the orders of MCs, we use counts of word patterns of length *k* (*k*≥1) to test if the two sequences are from the same MC of order *k*−1 as in [11]. The basic formulation of the problem can be described as follows. Let

$$ \mathbf{A}_{s} = A_{s,1} A_{s,2} \cdots \cdots A_{s, L_{s}},\ \ s = 1, 2, $$

where *L*_*s*_ is the length of the *s*-th sequence and *A*_*s,i*_, 1≤*i*≤*L*_*s*_ is the letter of the sequence at the *i*-th position.

To derive the likelihood ratio test, we assume that both sequences follow MCs of order *k*−1. The probability of the *s*-th sequence is

1 $$\begin{array}{@{}rcl@{}} P(\mathbf{A}_{s}) &=& \pi^{(s)}_{A_{s,1} A_{s,2} \cdots A_{s,k-1}} \prod_{i = k}^{L_{s}} t^{(s)}\left(A_{s, i-k+1} \cdots A_{s, i-1}, A_{s, i}\right)  \\ &=& \pi^{(s)}_{A_{s,1} A_{s,2} \cdots A_{s,k-1}} \prod_{\mathbf{w}} \left(t^{(s)}\left(\mathbf{w}^{-}, w_{k}\right)\right)^{N^{(s)}_{\mathbf{w}}},  \end{array} $$

where **w**=*w*_1_ *w*_2_⋯*w*_*k*_ is any word pattern of length *k*, **w** ^−^=*w*_1_ *w*_2_⋯*w*_*k*−1_ (the last letter is removed), $N^{(s)}_{\mathbf {w}}$ is the number of occurrences of word **w**, and *t* ^(*s*)^(**w** ^−^,*w*_*k*_) is the (*k*−1)-th order transition probability from **w** ^−^ to *w*_*k*_ in the *s*-th sequence, and *π* ^(*s*)^ is the initial distribution.

From this equation, it is easy to show that the maximum likelihood estimate of *t* ^(*s*)^(**w** ^−^,*w*_*k*_) is

$$ \hat{t}^{(s)}(\mathbf{w}^{-}, w_{k})= \frac{N^{(s)}_{\mathbf{w}}}{N^{(s)}_{\mathbf{w}^{-}}}. $$

Therefore, we can obtain the maximum likelihood for the *s*-th sequence $\hat {P}(\mathbf {A}_{s})$ by replacing *t* ^(*s*)^(**w** ^−^,*w*_*k*_) with $\hat {t}^{(s)}(\mathbf {w}^{-}, w_{k})$ in equation (1). The likelihood of both sequences under the alternative hypothesis *H*_1_ is

2 $${}  P_{1}\! = \prod_{s = 1}^{2}\! \hat{P}(\mathbf{A}_{s})\! = \prod_{s = 1}^{2} \pi^{(s)}_{A_{s,1} A_{s,2} \cdots A_{s,k-1}} \prod_{\mathbf{w}} \left(\hat{t}^{(s)}\left(\mathbf{w}^{-}, w_{k}\right)\right)^{N^{(s)}_{\mathbf{w}}}.  $$

Under the null hypothesis *H*_0_, the transition matrices for the two sequences are the same. Using the same argument as above, we can show that the maximum likelihood estimate of the common transition probability *t*(**w** ^−^,*w*_*k*_) is given by

$$ \hat{t}(\mathbf{w}^{-}, w_{k}) = \frac{N^{(-)}_{\mathbf{w}}}{ N^{(-)}_{\mathbf{w}^{-}}}, $$

where $N^{(-)}_{\mathbf {w}} = \sum _{s=1}^{2} N^{(s)}_{\mathbf {w}}$. Then the probability, *P*_0_, of both sequences can be estimated similarly as in Eq. (2). The log-likelihood ratio statistic is given by (ignoring the first *k*−1 bases in each sequence)

3 $$\begin{array}{@{}rcl@{}}  \log(P_{1}/P_{0}) & = & \sum_{s = 1}^{2} \sum_{w_{1} w_{2} \cdots w_{k-1}} \sum_{w_{k}} N^{(s)}_{\mathbf{w}} \log \left(\frac{\hat{t}^{(s)}(\mathbf{w}^{-}, w_{k})}{\hat{t}(\mathbf{w}^{-}, w_{k})} \right) \\ & = & \sum_{s = 1}^{2} \sum_{w_{1} w_{2} \cdots w_{k-1}} \sum_{w_{k}} N^{(s)}_{\mathbf{w}} \log \left(\frac{N^{(s)}_{\mathbf{w}} \times N^{(-)}_{\mathbf{w}^{-}}}{N^{(s)}_{\mathbf{w}^{-}} \times N^{(-)}_{\mathbf{w}}} \right)\\ \end{array} $$

The above statistic has an approximate *χ* ^2^-distribution as the lengths of both sequences become large [21, 22].

It has been shown that twice the log-likelihood ratio statistic has the same approximate distribution as the following *χ* ^2^-statistic [11] defined by

4 $$ S_{k} = \sum_{s = 1}^{2} \sum_{w_{1} w_{2} \cdots w_{k-1}} \sum_{w_{k}} \frac{\left(N^{(s)}_{\mathbf{w}} - N^{(s)}_{\mathbf{w}^{-}} N^{(-)}_{\mathbf{w}}/N^{(-)}_{\mathbf{w}^{-}} \right)^{2}}{N^{(s)}_{\mathbf{w}^{-}} N^{(-)}_{\mathbf{w}}/N^{(-)}_{\mathbf{w}^{-}}}.   $$

Since 2 log(*P*_1_/*P*_0_) and *S*_*k*_ are approximately equal, in our study, we use the measure *S*_*k*_ for sequence comparison.

To test if two independent identically distributed (i.i.d) sequences (*r*=0) have the same nucleotide frequencies, we set *k*=1, $N^{(s)}_{\mathbf {w}^{-}} = L_{s}, ~s = 1, 2$, $N^{(-)}_{\mathbf {w}^{-}} = L_{1} + L_{2},$ and *S*_1_ is calculated by

5 $$ S_{1} = \sum_{\mathbf{w}} \frac{L_{1} L_{2} \left(p_{\mathbf{w}}^{(1)} - p_{\mathbf{w}}^{(2)}\right)^{2}}{L_{1} p_{\mathbf{w}}^{(1)} + L_{2} p_{\mathbf{w}}^{(2)} },   $$

where **w** is a nucleotide and the summation is over all the nucleotides, $p_{\mathbf {w}}^{(s)} = N_{\mathbf {w}}^{(s)}/L_{s}$, and *L*_*s*_ is the length of the *s*-th sequence.

### Estimating the order of a MC sequence

We usually do not know the order, *r*, of the MC corresponding to each sequence and it needs to be estimated from the data. Several methods have been developed to estimate the order of a MC including those based on the Akaike information criterion (AIC) [23] and Bayesian information criterion (BIC) [24]. The AIC and BIC for a Markov sequence of length *L* are defined by

$$\begin{array}{@{}rcl@{}} \text{AIC}(k)&=&-2\sum_{\mathbf{w}\in\mathcal{A}^{k+1}}N_{\mathbf{w}}\log\frac{N_{\mathbf{w}}}{N_{\mathbf{w^{-}}}}+2(C-1)C^{k},\\ \text{BIC}(k)&=&-2\sum_{\mathbf{w}\in\mathcal{A}^{k+1}}N_{\mathbf{w}}\log\frac{N_{\mathbf{w}}}{N_{\mathbf{w^{-}}}}+(C-1)C^{k} \log (L-k+1), \end{array} $$

where *C* is the alphabet size. The estimators of the order of a Markov sequence based on AIC and BIC are given by

6 $$\begin{array}{@{}rcl@{}} \hat{r}_{\text{AIC}}=\arg\min_{k} AIC (k), \end{array} $$

7 $$\begin{array}{@{}rcl@{}} \hat{r}_{\text{BIC}}=\arg\min_{k} BIC (k).  \end{array} $$

Peres and Shields [25] proposed the following estimator for the order of a Markov chain

8 $$ \hat{r}_{PS}=\arg\max_{k} \left \{ \frac{\Delta^{k}}{\Delta^{k+1}} \right \}-1,   $$

where

$$\Delta^{k} = \max_{\mathbf{w}\in\mathcal{A}^{k}} |N_{\mathbf{w}}-E_{\mathbf{w}}|, $$

and $\mathcal {A}$ is the set of all alphabet and $E_{\mathbf {w}}=\frac { N_{\mathbf {-w}} N_{\mathbf {w-}} }{N_{\mathbf {-w-}}}$ is the expectation of word **w** estimated by a *k*−2-th order MC.

Based on similar ideas as in [25], Ren et al. [26] proposed several methods to estimate the order of a MC based on

$$T_{k} = \sum\limits_{\mathbf{w}\in\mathcal{A}^{k}}\frac{(N_{\mathbf{w}}-E_{\mathbf{w}})^{2}}{E_{\mathbf{w}}},\qquad \text{where}\quad E_{\mathbf{w}}=\frac{N_{\mathbf{-w}} N_{\mathbf{w-}}}{N_{\mathbf{-w-}}}. $$

The statistic *T*_*k*_ has an approximate *χ* ^2^-distribution with *df*_*k*_=(*C*−1)^2^ *C* ^*k*−2^ degrees of freedom when *k*≥*r*+2 [21, 22, 27, 28]. When *k*<*r*+2, *T*_*k*_ will be large if the sequence is long, while *T*_*k*_ should be moderate when *k*≥*r*+2. Based on this idea, we can estimate the order of the MC by

9 $$ \hat{r}_{T} = \arg\min_{k} \left \{\frac{T_{k+1}}{T_{k}} \right \}-1.   $$

Instead of using *T*_*k*_ directly, we can calculate the corresponding p-value

$$\begin{array}{@{}rcl@{}} p_{k}=P(T_{k} \geq t_{k})=P\left(\chi^{2}_{df_{k}} \geq t_{k}\right), \end{array} $$

where *t*_*k*_ is the observed value of *T*_*k*_ based on the long sequence. Since *t*_*k*_ is generally large when *k*≤*r*+1 and thus *p*_*k*_ should be small, while *p*_*k*_ is moderate when *k*≥*r*+2. Based on this idea, we can estimate the order of a MC by

10 $$ \hat{r}_{p}=\arg\min_{k} \left \{ \frac{\log(p_{k+1})}{\log(p_{k})} \right \}-1.   $$

It is also possible to estimate the order of a MC based on the counts of individual word patterns. Let

$$\begin{array}{@{}rcl@{}} Z_{\mathbf{w}}=\frac{N_{\mathbf{w}}-E_{\mathbf{w}}}{\hat{\sigma}_{\mathbf{w}}}, \end{array} $$

where $ \hat {\sigma }^{2}_{\mathbf {w}} = E_{\mathbf {w}} \left (1-\frac {N_{-\mathbf {w}}}{N_{-\mathbf {w}-}} \right) \left (1-\frac {N_{\mathbf {w}-}}{N_{-\mathbf {w}-}}\right)$ with $E_{\mathbf {w}} = \frac { N_{\mathbf {-w}} N_{\mathbf {w-}} }{N_{\mathbf {-w-}}}.$ It has been shown that, for every word **w**, *Z*_**w**_ is approximately normally distributed when *k*≥*r*+2. When the sequence is long, we expect *Z*_max_(*k*)= max**w**,|**w**|=*k*|*Z*_**w**_| to be large when *k*≤*r*+1, while it is moderate when *k*≥*r*+2. Similar to the ideas given above, we can estimate the order of the MC by

11 $$ \hat{r}_{Z} = \arg\min_{k} \left \{\frac{Z_{\max}(k+1)}{Z_{\max}(k)} \right \}-1.   $$

We are interested in knowing the power loss of the *χ* ^2^-statistic when any of the estimated orders of the two sequences are used for the comparison of MC sequences.

### Alignment-free comparison of two Markov sequences based on NGS reads

We then investigate the comparison of sequences based on NGS reads. We first extend the *χ* ^2^-statistic in Eq. (4) to be applicable to NGS reads. We then extend the methods for estimating the order of MC sequences for long sequences to be applicable to NGS reads. Finally, we study the optimal word length for genome comparison based on NGS reads and investigate the effect of sequence length, read length, distributions of reads along the genome, and sequencing errors on the power of the statistic.

### Alignment-free dissimilarity measures for comparing Markov sequences based on NGS reads

Next generation sequencing (NGS) technologies are widely used to sequence genomes. Instead of whole genome sequences, NGS data consists of short reads with lengths ranging from 100 bps to several hundred base pairs depending on the sequencing technologies. Since the reads are randomly chosen from the genomes, some regions can be sequenced multiple times while other regions may not be sequenced. The log-likelihood ratio statistic in Eq. (3) for long sequences cannot be directly extended to NGS reads because of the dependence of the overlapping reads. On the other hand, the *χ* ^2^-statistic in Eq. (4) depends only on word counts in the two sequences, and thus can be easily extended to NGS read data. We replace *N*_**w**_ in Eq. (4) by $N^{R}_{\mathbf {w}}$, the number of occurrences of word pattern **w** among the NGS reads, to obtain a new statistic,

12 $$\begin{array}{@{}rcl@{}} S_{k}^{R} &=& \sum_{s = 1}^{2} \sum_{w_{1} w_{2} \cdots w_{k-1}} \sum_{w_{k}} \frac{\left(N^{R(s)}_{\mathbf{w}} - N^{R(s)}_{\mathbf{w}^{-}} N^{R(-)}_{\mathbf{w}}/N^{R(-)}_{\mathbf{w}^{-}} \right)^{2}}{N^{R(s)}_{\mathbf{w}^{-}} N^{R(-)}_{\mathbf{w}}/N^{R(-)}_{\mathbf{w}^{-}}}, \\ \end{array} $$

13 $$\begin{array}{@{}rcl@{}} S_{1}^{R} &=& \sum_{\mathbf{w}} \frac{L_{1} L_{2} \left(p_{\mathbf{w}}^{R(1)} - p_{\mathbf{w}}^{R(2)}\right)^{2}}{L_{1} p_{\mathbf{w}}^{R(1)} + L_{2} p_{\mathbf{w}}^{R(2)} }. \end{array} $$

We will use $S_{k}^{R}$ to measure the dissimilarity between the two sequences.

### Estimating the order of a Markov sequence based on NGS reads

We next extend the estimators of the order of a MC in “Estimating the order of a MC sequence” subsection to NGS reads. The estimators *r*_AIC_ and *r*_BIC_ cannot be directly calculated because the likelihood of the reads is hard to calculate due to the potential overlaps among the reads. On the other hand, the other remaining estimators in “Estimating the order of a MC sequence” subsection, *r*_*PS*_, *r*_*S*_,*r*_*p*_, and *r*_*Z*_, depend only on the word counts and we can just replace *N*_**w**_ in these Eqs. by $N^{R}_{\mathbf {w}}$ for the NGS data. For simplicity of notation, we will continue to use the same notation as that in “Estimating the order of a MC sequence” subsection for the corresponding estimators. Similar to the study of long sequences, we investigate the power loss of the statistic $S_{k}^{R}$ when the estimated orders of the sequences are used to compare the power of $S_{k}^{R}$ when the true orders of the sequences are used.

## Results

### Optimal word length for the comparison of Markov sequences using the *χ*^2^-statistic

The following theorem gives the optimal word length for the comparison of two sequences using the *χ* ^2^-statistics given in Eqs. 4 and (5). The theoretical proof is given in the Additional file 1.

#### **Theorem 1**

Consider two Markov sequences of orders *r*_1_ and *r*_2_, respectively. We test the alternative hypothesis *H*_1_: the transition matrices of the two Markov sequences are different, versus the null hypothesis *H*_0_: the transition probability matrices are the same, using the *χ* ^2^-statistic in Eqs. (4) and (5). Then the power of the *χ* ^2^-statistic under the alternative hypothesis is maximized when the word length *k*= max{*r*_1_,*r*_2_}+1.

In the following, we present simulation results to show the power of the statistic *S*_*k*_ in Eqs. (4) and (5) for different values of sequence length and word pattern length. We simulated two Markov sequences **A**_1_ and **A**_2_ with different transition matrices and then calculated the distributions of the *χ* ^2^-statistic. We set the length of both sequences to be the same *L*: 10, 20 and 30 kbps, respectively, and started the sequences from the stationary distribution. We simulated MCs of first order and second order, respectively. Tables 1 and 2 show the transition probability matrices of (a) the first and (b) the second order transition matrices we used in the simulations. Here we present simulation results based on transition matrices from Tables 1 and 2 for simplicity. We also tried other transition matrices and the conclusions were the same.

**Table 1:** The transition probability matrix of the first order Markov chain in our simulation studies

	A	C	G	T
A	0.1	0.2	0.3	0.4
C	0.2	0.3	0.4	0.1
G	0.3	0.4	0.1	0.2
T	0.4	0.1	0.2	0.3

**Table 2:** The transition probability matrix of the second order Markov chain

	A	C	G	T
AA	0.1+ *α*_1_	0.2- *α*_1_	0.3+ *α*_2_	0.4- *α*_2_
AC	0.2+ *α*_1_	0.3- *α*_1_	0.4+ *α*_2_	0.1- *α*_2_
AG	0.3+ *α*_1_	0.4- *α*_1_	0.1+ *α*_2_	0.2- *α*_2_
AT	0.4+ *α*_1_	0.1- *α*_1_	0.2+ *α*_2_	0.3- *α*_2_
CA	0.1+ *β*_1_	0.2- *β*_1_	0.3+ *β*_2_	0.4- *β*_2_
CC	0.2+ *β*_1_	0.3- *β*_1_	0.4+ *β*_2_	0.1- *β*_2_
CG	0.3+ *β*_1_	0.4- *β*_1_	0.1+ *β*_2_	0.2- *β*_2_
CT	0.4+ *β*_1_	0.1- *β*_1_	0.2+ *β*_2_	0.3- *β*_2_
GA	0.1+ *γ*_1_	0.2- *γ*_1_	0.3+ *γ*_2_	0.4- *γ*_2_
GC	0.2+ *γ*_1_	0.3- *γ*_1_	0.4+ *γ*_2_	0.1- *γ*_2_
GG	0.3+ *γ*_1_	0.4- *γ*_1_	0.1+ *γ*_2_	0.2- *γ*_2_
GT	0.4+ *γ*_1_	0.1- *γ*_1_	0.2+ *γ*_2_	0.3- *γ*_2_
TA	0.1+ *δ*_1_	0.2- *δ*_1_	0.3+ *δ*_2_	0.4- *δ*_2_
TC	0.2+ *δ*_1_	0.3- *δ*_1_	0.4+ *δ*_2_	0.1- *δ*_2_
TG	0.3+ *δ*_1_	0.4- *δ*_1_	0.1+ *δ*_2_	0.2- *δ*_2_
TT	0.4+ *δ*_1_	0.1- *δ*_1_	0.2+ *δ*_2_	0.3- *δ*_2_

The parameters *α*_*i*_,*β*_*i*_,*γ*_*i*_,*δ*_*i*_, *i*=1,2, in Table 2 control the transition matrix of the second order MC. Note that if *α*_*i*_=*β*_*i*_=*γ*_*i*_=*δ*_*i*_, *i*=1,2, the MC will become a first order MC.

Under the null hypothesis, sequences **A**_1_ and **A**_2_ follow the same Markov model. So we set the transition matrices for both **A**_1_ and **A**_2_ to be Table 1. Under the alternative hypothesis, the two sequences are different and we set the transition matrix of sequence **A**_1_ to be from Table 1 and the transition matrix of sequence **A**_2_ to be from Table 2. We set the parameters of Table 2 to be (1) *α*_*i*_=*β*_*i*_=*γ*_*i*_=*δ*_*i*_=0.05, *i*=1,2, and (2) *α*_1_=*α*_2_=0.05,*β*_1_=*β*_2_=−0.05,*γ*_1_=*γ*_2_=0.03,*δ*_1_=*δ*_2_=−0.03. The former scenario corresponds to the situation that sequences **A**_1_ and **A**_2_ have different orders and the latter scenario corresponds to the situation that they both have first order but different transition matrices. We then calculated the dissimilarity measure between sequence **A**_1_ and **A**_2_ using the *χ* ^2^-statistic in Eq. (4).

We repeated the above procedures 2000 times to obtain an approximate distribution of *S*_*k*_ under the null hypothesis. We sorted the value of *S*_*k*_ in ascending order and took the 95% percentile as a threshold. Under the alternative hypothesis, the power is approximated by the fraction of times that *S*_*k*_ is above the threshold.

**Fig. 1:**
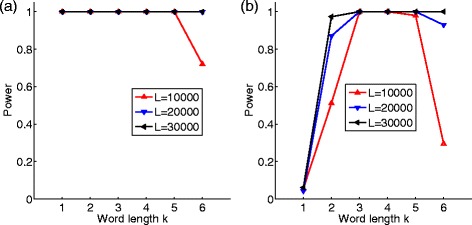
Relationship between the word length *k* and the power. The transition matrix of sequence **A**_1_ is from Table 1 and the transition matrix of sequence **A**_2_ is from Table 2 with the parameters being (**a**) *α*_*i*_=*β*_*i*_=*γ*_*i*_=*δ*_*i*_=0.05, *i*=1,2 for the first order MC and (**b**) *α*_1_=*α*_2_=0.05, *β*_1_=*β*_2_=−0.05,*γ*_1_=*γ*_2_=0.03,*δ*_1_=*δ*_2_=−0.03 for the second order MC

Figure 1 shows the relationship between the word size *k* and the power of *S*_*k*_ for long sequences of different lengths. It can be seen from the figure that the power of *S*_*k*_ is highest when the word length is *k*_optimal_= max{*r*_1_,*r*_2_}+1. When the word length is less than the optimal value, the power of *S*_*k*_ can be significantly lower. On the other hand, when the word length is slightly higher than the optimal word length, the power of *S*_*k*_ is still close to the optimal power. However, when the word length is too large, the power of *S*_*k*_ can be much lower.

**Fig. 2:**
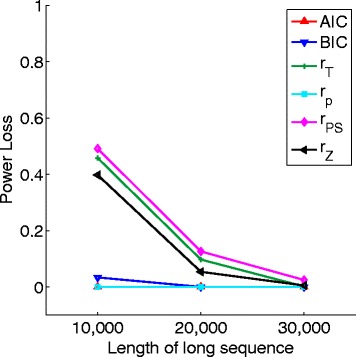
The power loss of the *χ* ^2^-statistic based on the estimated orders of the long sequences. A first order and a second order Markov long sequences are used

Given long sequences, the orders of the MCs are usually not known and have to be estimated from the data. We then studied how the power of *S*_*k*_ changes when the estimated orders of the sequences are used compared to the power when the true orders of the sequences are known. Let $\hat {r}_{1}$ and $\hat {r}_{2}$ be the estimated orders of sequences **A**_1_ and **A**_2_, respectively. We compared the power of $S_{\hat {k}}$ where $\hat {k} = \max \left \{ \hat {r}_{1}, \hat {r}_{2} \right \} + 1$ with that of *S*_*k*−optimal_ where *k*−optimal= max{*r*_1_,*r*_2_}+1. The power loss is defined as the difference between the power of *S*_*k*−optimal_ and that of $S_{\hat {k}}$. When both sequences are of first order, there was no power loss in our simulations. Figure 2 shows the power loss using different methods to estimate the orders of the sequences described in Eqs. (6) to (11) when the first sequence is of first order and the second sequence is of second order. There are significant differences among the various estimators when the sequence length is below 20 kbps. The power loss is minimal based on *r*_AIC_, *r*_BIC_, and *r*_*p*_ for all three sequence lengths from 10 to 30 kbps, indicating their good performance in estimating the true Markov order of the sequence. When the sequence length is long, e.g 30kpbs, the power loss is minimal for all the estimators across the sequence lengths simulated.

### Optimal word length for $S_{k}^{R}$ for the comparison of two Markov sequences with NGS data

The distribution of $S_{k}^{R}$ was not known previously. In this paper, we have the following theorem whose proof is given in the Additional file 1.

#### **Theorem 2**

Consider two Markov sequences with the same length *L* and Markov orders of *r*_1_ and *r*_2_, respectively. Suppose that they are sequenced using NGS with *M* reads of length *κ* for each sequence. Let $S_{k}^{R}$ be defined as in Eqs. (12) and (13). Suppose that each sequence can be divided into (not necessarily contiguous) regions with constant coverage *r*_*i*_ for the *i*-th region, so that every base is covered exactly *r*_*i*_ times. Let *L*_*is*_ be the length of the *i*-th region in the short read data for the *s*-th sequence and ${\lim }_{L\rightarrow \infty } L_{is}/L=f_{i},~s=1,2$. Then

1. Under the null hypothesis that the two sequences follow the same Markov chain, as sequence length *L* becomes large, $S_{k}^{R}/d$ is approximately *χ* ^2^-distributed with degrees of freedom *df*_*k*_=(*C*−1)*C* ^*k*−1^, where *C* is the alphabet size and
  14 $$ d=\frac{\sum_{i}r^{2}_{i}f_{i}}{\sum_{j}r_{j}f_{j}}.  $$
  In particular, under the Lander-Waterman model, the reads are randomly sampled from the long sequence so that the NGS reads follow a Poisson process with rate *λ*=*M* *κ*/*L*[29], for *r*_*i*_=*i*, *f*_*i*_=*λ* ^*i*^ exp(−*λ*)/*i*!, *d*=1+*λ*.
2. If we use $S_{k}^{R}$ to test whether the two sequences follow the same MC, under the alternative hypothesis, the power of $S_{k}^{R}$ is the highest when *k*= max{*r*_1_,*r*_2_}+1.

To illustrate the first part of Theorem 2, we simulated the distribution of $S^{R}_{k}$ under the null hypothesis. We assumed that both sequences are of order 1 with the transition probability matrix from Table 1. First, we generated MCs with length of *L*=10 and 20 kbps, respectively. The simulations of long sequences were the same as in “Optimal word length for the comparison of Markov sequences using the *χ*^2^-statistic” subsection. Second, we simulated NGS reads by sampling a varying number of reads from each sequence. The sampling of the reads was simulated as in [26,30]. The length of the reads was assumed to be a constant *κ*=200 bps and the number of reads *M* = 100 and 200 bps, respectively. The coverage of reads is calculated as *λ*=*M* *κ*/*L*. Two types of read distributions were simulated: (a) homogeneous sampling that the reads were sampled uniformly along the long sequence [29], and (b) heterogeneous sampling as in [31]. In heterogeneous sampling, we evenly divided the long genome sequences into 100 blocks. For each block, we sampled a random number independently from the gamma distribution *Γ*(1,20). The sampling probability for each position in the block is proportional to the chosen number.

Sequencing errors are present in NGS data. In order to see the effect of sequencing errors on the distribution of $S^{R}_{k}$, we simulated sequencing errors such that each base was changed to other three bases with equal probability 0.005.

Once the reads are generated, we then calculated $S^{R}_{k}$ between two NGS read data sets. In our simulation study, we fixed *k*=3 and the simulation process was repeated 2000 times for each combination of sequence length and number of reads (*L,M*) to obtain the approximate distribution of $S^{R}_{3}/d$, where *d* is given in Eq. (14).

**Fig. 3:**
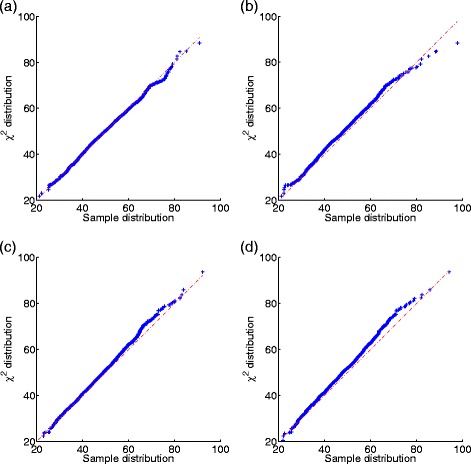
Q-Q plots of the 2000 $S^{R}_{3}/d$ scores v.s. 2000 scores sampled from a $\chi ^{2}_{48}$ distribution. The length of sequences *L* is 20kpbs and the number of reads *M* is 200. **a** homogeneous sampling without errors, **b** homogeneous sampling with errors, **c** heterogeneous sampling without errors, and **d** heterogeneous sampling with errors

Figure 3 shows the Q-Q (Quantile-Quantile) plots of the 2000 $S^{R}_{3}/d$ scores v.s. 2000 scores sampled from a $\chi ^{2}_{48}$ distribution, where the subscript 48 indicates the degrees of freedom of the *χ* ^2^ distribution. The constant *d* is 1+*λ* where *λ* denotes the coverage for homogeneous sampling; and *d* is calculated from Eq. (14) for heterogeneous sampling. It can be seen from the figure that the Q-Q plots center around the line *y*=*x* for both homogeneous and heterogeneous sampling without sequencing errors. These observations are consistent with part 1 of the Theorem 2. However, when sequence errors are present, the distribution of $S^{R}_{3}/d$ deviates slightly from $\chi ^{2}_{48}$.

We next studied how the power of $S_{k}^{R}$ changes with word length, sequence length, and sequencing errors. Here we show the results for the scenario that one sequence has first order and the other has second order. The results for the scenario that both sequences are of first order are given in the Additional file 1.

**Fig. 4:**
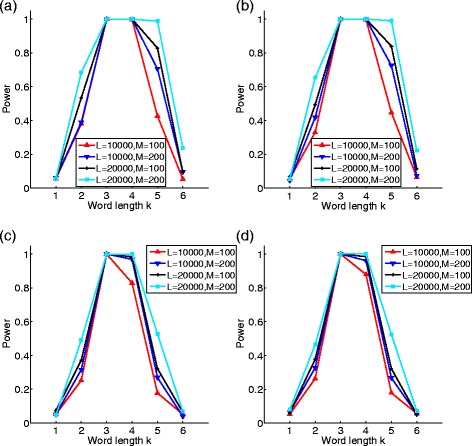
The relationship between the word length *k* and the power of $S_{k}^{R}$ based on NGS reads. The transition matrix of sequence **A**_1_ is from Table 1 and the transition matrix of **A**_2_ is from Table 2. The parameters of Table 2 are *α*_1_=*α*_2_=0.05,*β*_1_=*β*_2_=−0.05,*γ*_1_=*γ*_2_=0.03,*δ*_1_=*δ*_2_=−0.03. **a** homogeneous sampling without errors, **b** homogeneous sampling with errors, **c** heterogeneous sampling without errors, and **d** heterogeneous sampling with errors

The type I error was set at 0.05. Figure 4 shows the relationship between the word length *k* and the power of $S^{R}_{k}$ using NGS short reads for different sampling of the reads and with/without sequencing errors. Several conclusions can be derived. First, the power of $S^{R}_{k}$ is the highest when the word length *k*= max{*r*_1_,*r*_2_}+1. This is consistent with the result with long sequences. Second, sequencing errors can decrease the power of $S^{R}_{k}$. However, with the range of sequencing error rates of current technologies, the decrease in power is minimal. Third, the power of $S^{R}_{k}$ based on heterogeneous sampling of the reads is lower than that based on homogeneous sampling of the reads. Fourth, the power of $S^{R}_{k}$ increases with both sequence length *L* and number of reads *M* as expected.

**Fig. 5:**
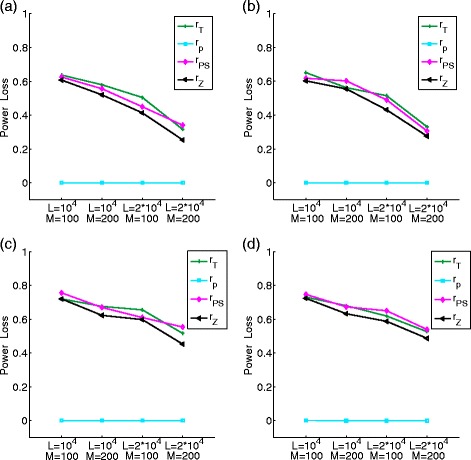
The power loss of $S_{k}^{R}$ based on different methods for estimating the order of Markov sequences based on NGS short reads. Panels are the same as in Fig. 4. **a** homogeneous sampling without errors, **b** homogeneous sampling with errors, **c** heterogeneous sampling without errors, and **d** heterogeneous sampling with errors

We then studied the effect on the power of $S_{k}^{R}$ using the estimated orders of the Markov sequences with NGS reads. We used a similar approach as in “Optimal word length for the comparison of Markov sequences using the *χ*^2^-statistic” subsection to study this problem except that we change long sequences to NGS reads. Figure 5 shows the results. It can be seen that the power loss is significant except when *r*_*p*_ was used to estimate the order of the sequences. In all the simulated scenarios, the power loss is very small when *r*_*p*_ is used to estimate the orders of Markov sequences. This result is consistent with the case of long sequences where *r*_*p*_ also performs the best.

## Applications to real data

### Searching for homologs of the human protein HSLIPAS

We used *S*_*k*_ to analyze the relationship of 40 sequences chosen from mammals, invertebrates, viruses, plants, etc. as in [32,33]. We used HSLIPAS human lipoprotein lipase (LPL) of length 1612 bps as the query sequence and searched for similar sequences from a library set containing 39 sequences with length from 322 to 14,121 bps. The relationships among all the 40 sequences are well understood. Among the 39 library sequences, 20 sequences are from the primate division of Genbank, classified as being related to HSLIPAS, and 19 sequences that are from the divisions other than the primate division of Genbank, classified as being not related.

Wu et al. [32] estimated the orders of the 40 sequences using Schwarz information criterion (SIC) [34] and found that 13 of them follow independent identically distributed (i.i.d) model (order = 0) and 27 of them follow a first order MC. We also used BIC and found the same results as SIC.

As in Wu et al. [32], we used *selectivity* and *sensitivity* to quantify the performance of the measure *S*_*k*_ for different values of *k*. First, we calculated the dissimilarity between HSLIPAS and each of the 39 sequences using *S*_*k*_ and then ranked the 39 sequences in ascending order according to the values of *S*_*k*_. The sequence closest to HSLIPAS is ranked as sequence 1, the sequence with the next shortest distance as sequence 2, etc. *Sensitivity* is defined as the number of HSLIPAS-related sequences found among the first 20 (1-20) library sequences. *Selectivity* is measured in terms of consecutive correct classifications [35], that is, starting from sequence 1, the total number of sequences are counted until the first non-HSLIPAS-related library sequence occurs. Thus, *selectivity* and *sensitivity* are scores from 0 to 20 and higher score means better performance on the real data set.

Table 3 shows the sensitivity and selectivity of *S*_*k*_ for different values of *k* from 1 to 6. It can be seen from Table 3 that *k*=2 yields the best result for both selectivity and sensitivity. Since about two thirds of the sequences have estimated order 1 and one third of the sequences have estimated order 0, the results are consistent with our conclusion.

**Table 3:** The selectivity and sensitivity of *S*_*k*_ for different word length *k* based on the comparison of HSLIPAS with 39 library sequences

Word length *k*	1	2	3	4	5	6
selectivity	7	11	10	7	3	1
sensitivity	13	17	16	13	12	9

### Comparison of CRM sequences in four mouse tissues

We also used *S*_*k*_ to analyze cis-regulatory module (CRM) sequences in four tissues from developing mouse embryo [36–38] as in Song et al. [4]. The four tissues we used are forebrain, heart, limb and midbrain, with the average sequence lengths to be 711, 688, 657, and 847 bps, respectively. For each tissue, we randomly chose 500 sequences from the CRM dataset to form the *positive* dataset. For each sequence in the positive dataset, we randomly selected a fragment from the mouse genome with the same length, ensuring a maximum of 30% repetitive sequences to form the *negative* dataset. Thus, we have a negative dataset containing another set of 500 sequences.

We calculated the pairwise dissimilarity of sequences within the positive and also the negative dataset using the *S*_*k*_ statistic with word length from 1-7. Then we merged the pairwise dissimilarity from the positive and negative datasets together. Sequences within the positive dataset should be closer than sequences within the negative dataset because the positive sequences should share some common CRMs. Therefore, we ranked the pairwise dissimilarity in ascending order and then predicted sequence pairs with distance smaller than a threshold as from the positive sequence pairs and otherwise we predicted them as coming from the negative pairs. For each threshold, we calculated the false positive rate and the true positive rate. Thus, by changing the threshold, we plotted the receiver operating characteristic (ROC) curve and calculated the area under the curve (AUC). For each tissue and each word length *k*, we repeated the above procedures 30 times.

We used BIC to estimate the MC orders of the sequences. The estimated orders of positive sequences for all four tissues are given in the Additional file 1. Almost all positive sequences in the positive dataset have estimated orders of 0 or 1. The results are similar for the negative sequences (data not shown).

**Fig. 6:**
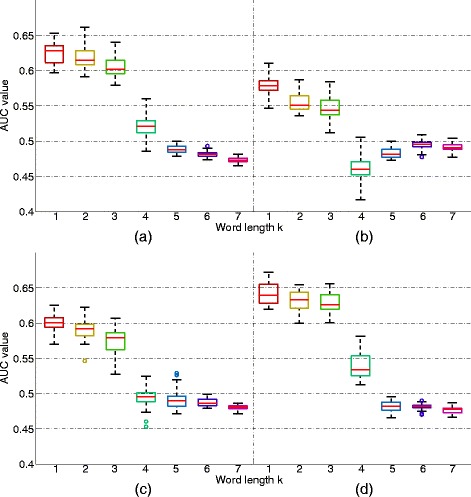
Boxplot of the AUC values for different word lengths *k*. For each *k* and each tissue, 30 AUC values based on 30 repeated experiments are shown. The subplots show results based on different tissues: **a** forebrain, **b** heart, **c** limb, and **d** midbrain

Figure 6 shows the relationship between the word length *k* and the AUC values in all four tissues using boxplot for the 30 replicates. It can be seen from the figure that the AUC values using word length 1-3 are much higher than that using word length 4-7. The AUC values when *k*=1 are slightly higher than that when *k*=2 and *k*=3. However, the differences are relatively small. The results are consistent in all four tissues. These results show that when the word length is close to the optimal word length based on our theoretical results, the AUC is generally higher than that when the word length is far away from the optimal word length based on our theoretical results.

## Discussion

In this paper, we investigated only the *χ* ^2^-statistic for alignment-free genome comparison and the optimality criterion is to maximize the power of the *χ* ^2^-statistic under the alternative hypothesis. Many other alignment-free genome comparison statistics are available as reviewed in [4,5]. The optimal word length we derived in this study may not be applicable to other statistics.

We assumed that the sequences of interest are Markov chains. Real molecular sequences do not exactly follow Markov chains and the sequences are also highly related. The relationship between the true evolution distance between the sequences and the pairwise *χ* ^2^-dissimilarity using the optimal word length needs to be further investigated. These are the topics for future studies.

## Conclusions

In this paper, we study the optimal word length when comparing two Markov sequences using word count statistics, in particular, the likelihood ratio statistic and the corresponding *χ* ^2^-statistic defined in Eq. (4). We showed theoretically and by simulations that the optimal word length is *k*= max{*r*_1_,*r*_2_}+1. When the orders of the sequences are not known and have to be estimated from the sequence data, we showed that the estimator *r*_*p*_ defined in Eq. (10) and the estimator *r*_AIC_ defined in Eq. (6) have the best performance, followed by *r*_BIC_ defined in Eq. (7) based on long sequences. We then extended these studies to NGS read data and found that the conclusions about the optimal word length continue to hold. It was also shown that if we use *r*_*p*_ defined in Eq. (10) to estimate the orders of the Markov sequences based on NGS reads $\hat {r}_{p1}$ and $\hat {r}_{p2}$, respectively, and then compare the sequences using $S_{\hat {k}-\text {optimal}}$, with $\hat {k}-\text {optimal} = \max \{ \hat {r}_{p1}, \hat {r}_{p2} \}+1 $, the power loss is minimal. These conclusions are not significantly changed by sequencing errors. Therefore, our studies provide guidelines on the optimal choice of word length for alignment-free genome comparison using the *χ* ^2^-statistic.

## Additional file

Additional file 1 Supplementary Materials. Proofs of Theorem 1 and 2, simulation results for the comparison of two first order Markov sequences based on NGS reads and estimated orders of positive sequences in four mouse tissues. (PDF 274 kb)
