# Supplementary material for: Optimal choice of word length when comparing two Markov sequences using a χ2-statistic
Source: BMC Genomics. 2017 Oct 3;18(Suppl 6):732. doi: 10.1186/s12864-017-4020-z (PMC5629589; doi:10.1186/s12864-017-4020-z)
Supplement: Supplementary file 1 — Supplementary Materials. Proofs of Theorem 1 and 2, simulation results for the comparison of two first order Markov sequences based on NGS reads and estimated orders of positive sequences in four mouse tissues. (PDF 274 kb) [file 12864_2017_4020_MOESM1_ESM.pdf]

RESEARCH

Optimal choice of word length when comparing two Markov sequences using a χ^2-statistic: supplementary materials

Xin Bai[1], Kujin Tang[2], Jie Ren[2], Michael Waterman[1,2] and Fengzhu Sun[1,2*]

*Correspondence: fsun@usc.edu
[1]Centre for Computational Systems Biology, School of Mathematical Sciences, Fudan University , Shanghai, China
[2]Molecular and Computational Biology Program, University of Southern California, Los Angeles, California, USA
Full list of author information is available at the end of the article

1 Appendix

1.1 Proof of Theorem 1

Let r_1 and r_2 $(r_1 \leq r_2)$ be the orders of sequences $\mathbf{A}_1$ and $\mathbf{A}_2$, respectively. If $k < r_2 + 1$, even under the null hypothesis that the two sequences are from the same MC, the statistic S_k is large and does not follow a χ^2-distribution. For a given type I error α, the threshold for rejecting the null hypothesis should be very big. Therefore, the power of S_k under the alternative hypothesis should be low.

When $k \geq r_2 + 1$, S_k will be χ^2-distributed with $C^{k-1}(C-1)$ degrees of freedom under the null hypothesis, where C is the size of the alphabet. Under the alternative hypothesis, S_k will have a non-central χ^2-distribution with non-centrality parameter that can be computed as follows. Without loss of generality, we assume that the sequences have the same length L. Note from the law of large numbers for MCs [1, 2],

$$\begin{aligned}
\lim_{L\to\infty} N_{\mathbf{w}}^{(s)}/L &= \pi^{(s)}(\mathbf{w}), \\
\lim_{L\to\infty} N_{\mathbf{w}}^{(s)}/N_{\mathbf{w}^-}^{(s)} &= t^{(s)}(\mathbf{w}^-, w_k), \\
\lim_{L\to\infty} N_{\mathbf{w}}^{(-)}/N_{\mathbf{w}^-}^{(-)} &= \frac{\sum_{s=1}^{2} \pi_{\mathbf{w}^-}^{(s)} t^{(s)}(\mathbf{w}^-, w_k)}{\sum_{s=1}^{2} \pi_{\mathbf{w}^-}^{(s)}} = \bar{t}(\mathbf{w}^-, w_k).
\end{aligned}$$

For simplicity of notation, we let $\mathbf{u}$ denote word of length $k-1$. Therefore, we can obtain the non-centrality parameter of S_k as

$$L \times \phi_k^2 = L \sum_{\mathbf{u}} \sum_{c=1}^{C} \phi_k^2(\mathbf{u}, c), \tag{1}$$

where the first summation is over all words of length $k-1$ and

$$\phi_k^2(\mathbf{u}, c) = \frac{\pi^{(1)}(\mathbf{u})\pi^{(2)}(\mathbf{u})(t^{(1)}(\mathbf{u}, c) - t^{(2)}(\mathbf{u}, c))^2}{\sum_{s=1}^{2} \pi^{(s)}(\mathbf{u}) t^{(s)}(\mathbf{u}, c)}, \tag{2}$$

$t^{(s)}(\mathbf{u}, c)$ is the transition probability from $(k-1)$-word $\mathbf{u}$ to the letter c, and $\pi^{(s)}(\mathbf{u})$ is the stationary probability of $(k-1)$-word $\mathbf{u}$ for the s-th sequence. Since $\pi^{(s)}(\mathbf{u})$

is a stationary distribution, we have for any base c^* and a $(k-1)$-word $\mathbf{u}$, we have the following recursive equation

$$\pi^{(s)}(\mathbf{u}c^*) = \sum_{c=1}^{C} \pi^{(s)}(c\mathbf{u})t^{(s)}(c\mathbf{u}, c^*), \quad s = 1, 2. \tag{3}$$

Next we show that

$$\phi_k^2(\mathbf{u}, c^*) \geq \sum_{c=1}^{C} \phi_{k+1}^2(c\mathbf{u}, c^*), \text{ for all } (k-1) - \text{word } \mathbf{u} \text{ and letter } c^*, \tag{4}$$

where $c\mathbf{u}$ is a k-word with the first letter c followed by a $(k-1)$-word $\mathbf{u}$ and the sum is over all the alphabet. The above inequality indicates that the non-centrality parameter decreases with respect to k.

Without loss of generality, we just prove the case that $\mathbf{u}$ is a given $(k-1)$-word $\mathbf{I}_{k-1} = 11\cdots 1,\ u_i = 1,\ \forall 1 \leq i \leq k-1$ and $c^* = 1$. The proof is similar for other word patterns $\mathbf{u}$ and letter c^*.

For simplicity of notation, let

$$\begin{aligned} x_c &= t^{(1)}(c\mathbf{I}_{r_1-1}, 1), & y_c &= t^{(2)}(c\mathbf{I}_{r_2-1}, 1), \\ \mu_c &= \pi^{(1)}(c\mathbf{I}_{r_1-1}), & \nu_c &= \pi^{(2)}(c\mathbf{I}_{r_2-1}). \end{aligned}$$

Since sequences $\mathbf{A}_1$ and and $\mathbf{A}_2$ are Markov sequences of orders r_1 and r_2, respectively, we have for any $k \geq \max\{r_1, r_2\}$+1,

$$\begin{aligned} t^{(1)}(\mathbf{I}_{k-1}, 1) &= x_1, & t^{(2)}(\mathbf{I}_{k-1}, 1) &= y_1, \\ \pi^{(1)}(\mathbf{I}_{k-1}) &= \mu_1 x_1^{k-r_1-1}, & \pi^{(2)}(\mathbf{I}_{k-1}) &= \nu_1 y_1^{k-r_2-1}. \end{aligned}$$

Therefore,

$$\phi_k^2(\mathbf{I}_{k-1}, 1) = \frac{\mu_1 x_1^{k-r_1-1} \nu_1 y_1^{k-r_2-1} (x_1 - y_1)^2}{\mu_1 x_1^{k-r_1} + \nu_1 y_1^{k-r_2}}.$$

We also have

$$\begin{aligned} t^{(1)}(c\mathbf{I}_{k-1}, 1) &= x_1, & t^{(2)}(c\mathbf{I}_{k-1}, 1) &= y_1, \\ \pi^{(1)}(c\mathbf{I}_{k-1}) &= \mu_c x_c x_1^{k-r_1-1}, & \pi^{(2)}(c\mathbf{I}_{k-1}) &= \nu_c y_c y_1^{k-r_2-1}. \end{aligned}$$

Thus,

$$\sum_{c=1}^{C} \phi_{k+1}^2(c\mathbf{I}_{k-1}, 1) = \sum_{c=1}^{C} \frac{\mu_c x_c x_1^{k-r_1-1} \nu_c y_c y_1^{k-r_2-1} (x_1 - y_1)^2}{\mu_c x_c x_1^{k-r_1} + \nu_c y_c y_1^{k-r_2}}.$$

Both $\phi_k^2(\mathbf{I}_{k-1}, 1)$ and $\sum_{c=1}^{C} \phi_{k+1}^2(c\mathbf{I}_{k-1}, 1)$ have a common factor $x_1^{k-r_1-1} y_1^{k-r_2-1} (x_1 - y_1)^2$. Therefore, we only need to prove the following inequality

$$\frac{\mu_1 \nu_1}{\mu_1 x_1^{k-r_1} + \nu_1 y_1^{k-r_2}} \geq \sum_{c=1}^{C} \frac{\mu_c x_c \nu_c y_c}{\mu_c x_c x_1^{k-r_1} + \nu_c y_c y_1^{k-r_2}}. \tag{5}$$

From equation (3), we have

$$\mu_1 = \sum_{c=1}^{C} \mu_c x_c, \quad \nu_1 = \sum_{c=1}^{C} \nu_c y_c.$$

The inequality (5) follows from Jensen's inequality [3] because the function

$$f(\mu_1, \nu_1) = \frac{\mu_1 \nu_1}{\mu_1 x_1^{k-r_1} + \nu_1 y_1^{k-r_2}} \tag{6}$$

is a two dimensional concave function [4].

□

1.2 Proof of Theorem 2

Proof of part 1. Under the null hypothesis that the two sequences follow the same Markov chain, the stationary distribution and the transition probability matrices are the same for the two sequences. Let $\pi(\mathbf{w})$ and $t(\mathbf{w}, c)$ be the common stationary probability and transition probability, respectively. For NGS short read data, let $N_{\mathbf{w}}^{R(s)}$ be the number of occurrences of the word $\mathbf{w}$ in the s-th short read data ($s = 1, 2$), where the superscript R refers to the "read" data. Denote $D_{\mathbf{w}}^{R(s)} = N_{\mathbf{w}^-}^{R(s)} N_{\mathbf{w}}^{R(-)} / N_{\mathbf{w}^-}^{R(-)}$. Then S_k^R can be re-written as

$$S_k^R = \sum_{s=1}^{2} \sum_{w_1 w_2 \cdots w_{k-1}} \sum_{w_k} \frac{(N_{\mathbf{w}}^{R(s)} - D_{\mathbf{w}}^{R(s)})^2}{D_{\mathbf{w}}^{R(s)}}. \tag{7}$$

Suppose that each genome can be divided into (not necessarily contiguous) regions with constant coverage r_i for the i-th region, so that every base is covered exactly r_i times. Let L_{is} be the length of the i-th region in the s-th short read data and $\lim_{L\to\infty} L_{is}/L = f_i > 0, \ s = 1, 2$.

Let $N_{\mathbf{w}}^{R(s)}(i)$ denote the the number of reads mapped to the the i-th region and $N_{\mathbf{w}}^{(s)}(i)$ denote the number of occurrences of $\mathbf{w}$ in the i-th region of the s-th genome. Let $D_{\mathbf{w}}^{R(s)}(i)$ be defined similarly as $D_{\mathbf{w}}^{R(s)}$ for the i-th region and $D_{\mathbf{w}}^{(s)} = N_{\mathbf{w}^-}^{(s)} N_{\mathbf{w}}^{(-)} / N_{\mathbf{w}^-}^{(-)}$. It is clear that $N_{\mathbf{w}}^{R(s)} = \sum_i N_{\mathbf{w}}^{R(s)}(i) = \sum_i r_i N_{\mathbf{w}}^{(s)}(i)$. From the law of large numbers for MCs[1, 2], we have for each region i and each sequence s

$$\lim_{L\to\infty} \frac{N_{\mathbf{w}^-}^{(s)}(i)}{L_{is}} = \pi(\mathbf{w}^-), \tag{8}$$

$$\lim_{L\to\infty} \frac{N_{\mathbf{w}}^{(-)}}{N_{\mathbf{w}^-}^{(-)}} = t(\mathbf{w}^-, w_k). \tag{9}$$

So we have approximately,

$$\begin{aligned} D_{\mathbf{w}}^{(s)}(i) &\approx L_{is} \pi(\mathbf{w}^-) t(\mathbf{w}^-, w_k) = L_{is} \pi(\mathbf{w}), \\ D_{\mathbf{w}}^{R(s)} &\approx \sum_i r_i D_{\mathbf{w}}^{(s)}(i). \end{aligned}$$

We assume that word length is small compared to read length κ, and hence the edge effects are small. Similar to Ren et al. [5], we can show that

$$\lim_{L\to\infty} \frac{D_{\mathbf{w}}^{R(s)} - \sum_i D_{\mathbf{w}}^{R(s)}(i)}{\sqrt{D_{\mathbf{w}}^{R(s)}}} = 0. \tag{10}$$

for any word $\mathbf{w}$.

With equation (10), S_k^R has approximately the same distribution as

$$\begin{aligned} S_k^{R,*} &= \sum_{s=1,2} \sum_{w_1 w_2 \cdots w_{k-1}} \sum_{w_k} \frac{(N_{\mathbf{w}}^{R(s)} - \sum_i D_{\mathbf{w}}^{R(s)}(i))^2}{D_{\mathbf{w}}^{R(s)}} \\ &= \sum_{s=1,2} \sum_{w_1 w_2 \cdots w_{k-1}} \sum_{w_k} \left(\sum_i \frac{N_{\mathbf{w}}^{R(s)}(i) - D_{\mathbf{w}}^{R(s)}(i)}{\sqrt{D_{\mathbf{w}}^{R(s)}}} \right)^2 \\ &= \sum_{s=1,2} \sum_{w_1 w_2 \cdots w_{k-1}} \sum_{w_k} \left(\sum_i \frac{N_{\mathbf{w}}^{(s)}(i) - D_{\mathbf{w}}^{(s)}(i)}{\sqrt{D_{\mathbf{w}}^{(s)}(i)}} \sqrt{\frac{r_i D_{\mathbf{w}}^{R(s)}(i)}{D_{\mathbf{w}}^{R(s)}}} \right)^2 \\ &= \sum_{s=1,2} \sum_{w_1 w_2 \cdots w_{k-1}} \sum_{w_k} \left(\sum_i W_i^{(s)} \frac{N_{\mathbf{w}}^{(s)}(i) - D_{\mathbf{w}}^{(s)}(i)}{\sqrt{D_{\mathbf{w}}^{(s)}(i)}} \right)^2 \end{aligned}$$

where

$$W_i^{(s)} = \sqrt{\frac{r_i D_{\mathbf{w}}^{R(s)}(i)}{D_{\mathbf{w}}^{R(s)}}} \approx \sqrt{\frac{r_i^2 f_i}{\sum_j r_j f_j}}.$$

The remaining steps of the proof are similar as that in [5] and are not presented here. □

Proof of part 2. We have shown that under the null hypothesis, S_k^R/d follows an approximate χ^2-distribution with $C^{k-1}(C-1)$ degrees of freedom. Let $\Delta = \sum_i r_i f_i$. Under the alternative hypothesis, for any word $\mathbf{w}$, we have

$$N_{\mathbf{w}}^{R(s)} \approx \sum_i r_i f_i N_{\mathbf{w}}^{(s)} = \Delta N_{\mathbf{w}}^{(s)}.$$

Similar as in subsection 1.1, we can obtain the non-centrality parameter of S_k^R as

$$L \times \phi_k^{R,2} = L \sum_{\mathbf{u}} \sum_{c=1}^{C} \phi_k^{R,2}(\mathbf{u}, c),$$

where the first summation is over all words of length $k-1$ and

$$\phi_k^{R,2}(\mathbf{u}, c) = \frac{\Delta \pi^{(1)}(\mathbf{u}) \pi^{(2)}(\mathbf{u}) (t^{(1)}(\mathbf{u}, c) - t^{(2)}(\mathbf{u}, c))^2}{\sum_{s=1}^{2} \pi^{(s)}(\mathbf{u}) t^{(s)}(\mathbf{u}, c)}, \tag{11}$$

This part follows from the proof of Theorem 1. □

1.3 Simulation results for the comparison of two first order Markov sequences based on NGS reads

We did simulation studies to compare the power of S_k^R using NGS reads when both sequences are of first order. The results are shown in **Figure** S1.

We also studied the power loss of S_k^R when the estimated orders of the sequences were used using NGS reads. There was no power loss under this scenario.

1.4 Estimated orders of positive sequences in four mouse tissues

We used BIC to estimate the MC orders of the positive sequences in four mouse tissues. For each tissue, we conduct 30 replicates randomly. Except for some replicates for tissue limb having orders $r \geq 2$, all positive sequences have order 0 and order 1. The fractions of positive sequences having different MC orders are listed in **Table S1**.

Table S1: Fractions of positive sequences with different estimated orders for each replicate and each tissue.

Replicate	Forebrain		heart		limb			midbrain	
	$r=0$	$r=1$	$r=0$	$r=1$	$r=0$	$r=1$	$r \geq 2$	$r=0$	$r=1$
1	0.436	0.564	0.416	0.584	0.476	0.524	0	0.382	0.618
2	0.426	0.574	0.418	0.582	0.450	0.548	0.002	0.040	0.060
3	0.442	0.558	0.402	0.598	0.486	0.508	0.006	0.428	0.572
4	0.440	0.560	0.430	0.570	0.440	0.546	0.014	0.392	0.608
5	0.466	0.534	0.388	0.612	0.452	0.544	0.004	0.438	0.562
6	0.492	0.508	0.422	0.578	0.464	0.524	0.010	0.394	0.606
7	0.420	0.580	0.420	0.580	0.470	0.518	0.012	0.406	0.594
8	0.408	0.592	0.418	0.582	0.446	0.544	0.010	0.410	0.590
9	0.392	0.608	0.392	0.608	0.452	0.546	0.002	0.414	0.586
10	0.420	0.580	0.388	0.612	0.440	0.560	0	0.394	0.606
11	0.414	0.586	0.428	0.572	0.450	0.542	0.008	0.392	0.608
12	0.394	0.606	0.452	0.548	0.440	0.560	0	0.390	0.610
13	0.414	0.586	0.420	0.580	0.418	0.582	0	0.378	0.622
14	0.446	0.554	0.430	0.570	0.440	0.554	0.006	0.356	0.644
15	0.390	0.610	0.418	0.582	0.450	0.500	0.050	0.384	0.616
16	0.420	0.580	0.412	0.588	0.412	0.584	0.004	0.380	0.620
17	0.416	0.584	0.406	0.594	0.448	0.526	0.026	0.416	0.584
18	0.428	0.572	0.420	0.580	0.470	0.528	0.002	0.396	0.604
19	0.428	0.572	0.420	0.580	0.424	0.522	0.054	0.402	0.598
20	0.444	0.556	0.414	0.586	0.422	0.578	0	0.404	0.596
21	0.402	0.598	0.396	0.604	0.458	0.538	0.004	0.448	0.552
22	0.420	0.580	0.400	0.600	0.446	0.554	0	0.398	0.602
23	0.428	0.572	0.422	0.578	0.432	0.524	0.044	0.388	0.612
24	0.418	0.592	0.424	0.576	0.418	0.572	0.010	0.374	0.626
25	0.428	0.572	0.396	0.604	0.406	0.558	0.036	0.422	0.578
26	0.436	0.564	0.412	0.588	0.462	0.532	0.006	0.418	0.582
27	0.426	0.574	0.410	0.590	0.478	0.488	0.034	0.398	0.602
28	0.458	0.542	0.428	0.572	0.476	0.482	0.042	0.406	0.594
29	0.408	0.592	0.428	0.572	0.440	0.530	0.030	0.412	0.588
30	0.454	0.546	0.410	0.590	0.444	0.548	0.008	0.424	0.576

Author details

[1]Centre for Computational Systems Biology, School of Mathematical Sciences, Fudan University , Shanghai, China. [2]Molecular and Computational Biology Program, University of Southern California, Los Angeles, California, USA.

References

1. Billingsley, P.: Statistical Inference for Markov Processes vol. 2. University of Chicago Press, Chicago (1961)
2. Billingsley, P.: Statistical methods in Markov chains. The Annals of Mathematical Statistics **32**(1), 12–40 (1961)

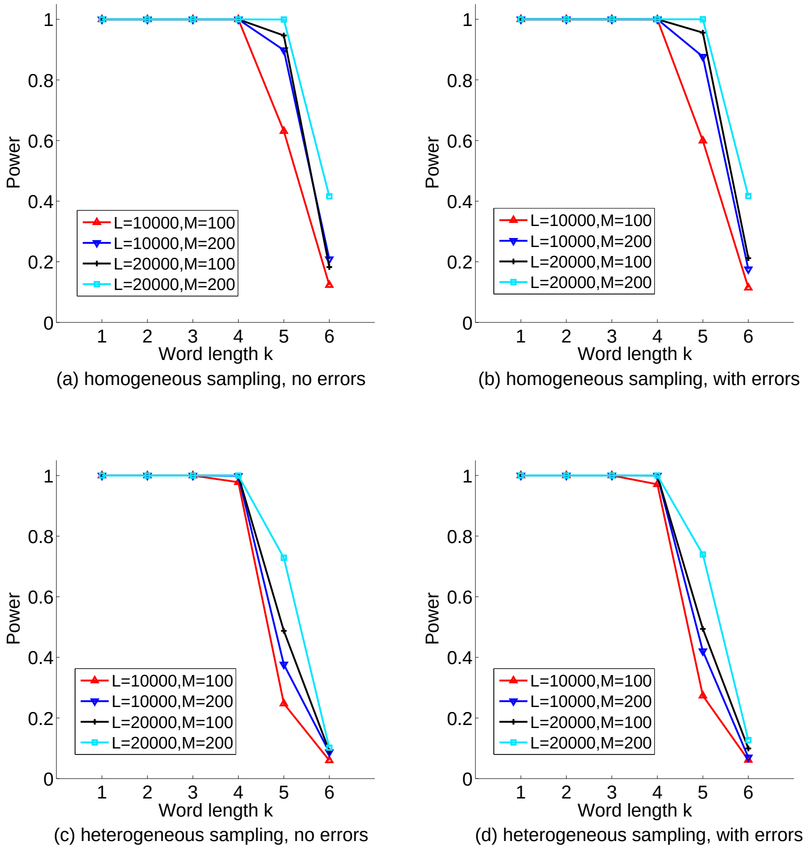

Figure S1: The relationship between the word size k and the power of S_k^R based on NGS reads. The transition matrix of sequence $\mathbf{A}_1$ is from **Table 1** and the transition matrix of $\mathbf{A}_2$ is from **Table 2** with parameters being $\alpha_i = \beta_i = \gamma_i = \delta_i = 0.05,\ i = 1, 2.$ (a) homogeneous sampling without errors, (b) homogeneous sampling with errors, (c) heterogeneous sampling without errors, and (d) heterogeneous sampling with errors.

3. Jensen, J.L.W.V.: Sur les fonctions convexes et les inégalités entre les valeurs moyennes. Acta Mathematica **30**(1), 175–193 (1906)
4. Boyd, S., Vandenberghe, L.: Convex optimization, pp. 67–71. Cambridge University Press, New York (2004)
5. Ren, J., Song, K., Deng, M., Reinert, G., Cannon, C.H., Sun, F.: Inference of Markovian properties of molecular sequences from NGS data and applications to comparative genomics. Bioinformatics **32**(7), 993–1000 (2016)
